# Community informed recommendations for a campaign to promote awareness of long-acting injectable PrEP among Latino men who have sex with men

**DOI:** 10.1371/journal.pone.0345564

**Published:** 2026-03-20

**Authors:** Ronald A. Brooks, Omar Nieto, Martin Santillan, Elena P. Rosenberg-Carlson, Jose R. Segura-Bermudez, Dilara K. Üsküp

**Affiliations:** 1 Department of Family Medicine, University of California, Los Angeles, California, United States of America; 2 Center for HIV Identification, Prevention, and Treatment Services (CHIPTS), University of California, Los Angeles, California, United States of America; 3 Department of Research and Evaluation, Bienestar Human Services, Los Angeles, California, United States of America; 4 David Geffen School of Medicine, University of California, Los Angeles, California, United States of America; 5 Department of Internal Medicine, Charles R. Drew University of Medicine and Science, Los Angeles, California, United States of America; 6 UCLA-CDU Center for AIDS Research (CFAR), Los Angeles, California, United States of America; Riphah International University, PAKISTAN

## Abstract

**Background:**

Across the United Sates, Latino men who have sex with men (LMSM) face disproportionately high HIV risk. Although pre-exposure prophylaxis (PrEP) effectively reduces HIV risk, numerous barriers limit PrEP access among LMSM. Culturally tailored public health campaigns provide one potential strategy for increasing access, awareness, and use of PrEP, including the newest long-acting injectable PrEP (LAI PrEP) formulations. The purpose of the study was to develop community-informed recommendations for a culturally responsive LAI PrEP awareness campaign designed for LMSM.

**Methods:**

Between June 2023 to March 2024, thirty LMSM participated in four virtual focus groups conducted in Los Angeles, CA. LMSM were recruited through a larger LMSM-centered HIV prevention intervention. In the focus groups, participants shared their perspectives on campaign messaging, imagery, tone, and accessibility. Coding reliability thematic analysis was used to analyze the data.

**Results:**

Findings suggest that a LAI PrEP campaign for LMSM should: provide basic information about the strategy; highlight its benefits; incorporate humorous and sex-positive messaging or imagery; communicate the seriousness of HIV without stigmatizing, blaming, or scaring LMSM; incorporate important Latine cultural values; and include Latine celebrities, public figures, and everyday individuals as messengers. In addition, there were mixed opinions regarding the use of a syringe in the imagery, with some participants advocating against its use to avoid fear- and pain-based associations with injections. Finally, participants believed that a LAI PrEP campaign should be made accessible to LMSM by providing materials in both English and Spanish and placing physical advertisements in neighborhoods predominately occupied by Latine people.

**Conclusions:**

Our findings offer several practical recommendations for developing a LAI PrEP awareness campaign for LMSM that can be used by health departments, community-based organizations, and others. Incorporating community insights is crucial to ensure that a campaign resonates with and meets the needs of the LMSM community.

## Introduction

While rates of HIV infection have decreased over time, men who have sex with men (MSM) continue to comprise the largest proportion of annual HIV cases in the United States. In 2022, MSM comprised 67% of all new HIV infections in the U.S. [1]. In addition, Latino MSM (LMSM) were disproportionately impacted by HIV relative to White MSM, with LMSM accounting for 37% of new infections compared to 23% among White MSM [1]. Greater efforts are needed to expand access to effective HIV prevention options among LMSM, including the newly available long-acting injectable pre-exposure prophylaxis (LAI PrEP) formulations.

First approved by the US Food and Drug Administration (FDA) in 2012, PrEP is highly effective in preventing acquisition of HIV in persons at risk of HIV infection and is a key prevention strategy in the Ending the HIV Epidemic in the US (EHE) initiative [2,3]. Since its approval, use of oral-based PrEP for HIV prevention has been increasing. Unfortunately, the rate of adoption has been suboptimal among racial/ethnic minority populations [4,5]. In 2021, oral-based PrEP prescriptions were highest among White individuals (78%) and lowest for Latine (21%) and Black (11%) persons with an indication for PrEP [4]. Similar low adoption rates have been seen among minoritized MSM. In the 2017 National HIV Behavioral Surveillance study, PrEP use was lowest among Black and Latino MSM (26% and 30%, respectively) compared with 42% among White MSM [5]. Common social barriers to PrEP use include racism, xenophobia, homophobia, medical mistrust, and HIV stigma in healthcare settings [6,7]. In addition, structural barriers such as cost of HIV prevention services, lack of health insurance, limited healthcare access, and difficulty navigating healthcare systems have also been shown to impede PrEP access [7–10]. Altogether, these social and structural factors contribute to suboptimal rates of PrEP adoption among minoritized MSM, including LMSM.

In 2021, the FDA approved Apretude as the first long-acting injectable formulation of PrEP. As a new biomedical HIV prevention option, LAI PrEP is in the initial stages of rollout, and currently there are no population-level data available on its uptake among populations at risk for HIV. However, given the historical trend of low adoption rates of oral-based PrEP among racial/ethnic minority populations [5,11,12], we might expect to see a similar pattern emerge with LAI PrEP. For any formulation of PrEP to achieve its full public health potential, its uptake must be proportionate to risk levels of vulnerable populations [11]. However, increasing uptake requires a concerted effort to make PrEP more accessible, and to inform and build awareness among racial/ethnic minority populations, including LMSM, of the latest innovations in HIV prevention.

Use of LAI PrEP over oral PrEP may be particularly beneficial for LMSM due to the high levels of homophobia experienced in Latine communities and families [13–16]. By avoiding the presence of pill or medication bottles, LAI PrEP offers LMSM a greater level of discretion, which may protect them from unintentionally being outed as gay and minimize the stigma associated with taking PrEP. In addition, LAI PrEP offers LMSM greater convenience when compared to the oral PrEP option (i.e., individuals see their physician once every two months for an injection versus having to remember to take a daily pill), which may enhance uptake and adherence [13–17].

Public health campaigns offer one method for increasing awareness and use of new HIV prevention options among priority populations. Prior research has demonstrated that health campaigns, particularly when community-informed, can be effective in increasing condom use, HIV & STI testing, PrEP awareness, and willingness to initiate oral-based PrEP among diverse populations of MSM [18–23]. However, LMSM have not been the explicit focus of this prior work, despite the increasing prevalence of HIV within the population. In contrast, multiple studies have focused on eliciting opinions from Black MSM, specifically, to both develop and evaluate the content of oral pill-based PrEP campaigns [20–22,24–28]. Unfortunately, the lack of focus on LMSM may result in the development of HIV prevention messaging that does not resonate with nor have the intended impact on this population. In addition, no studies to-date have aimed to work with HIV prevention priority populations, such as LMSM, to elicit community-informed recommendations for a campaign to promote awareness of LAI PrEP among this population.

This study sought to fill these gaps in the extant research literature by eliciting suggestions from LMSM regarding culturally centered messaging and imagery that could be used to inform the development of a LAI PrEP awareness campaign to engage and resonate with LMSM. In doing so, this study aims to support the EHE goals of reducing the number of incident HIV infections in the United States by 75 percent in 2025 and 90 percent in 2030 [29]. Of note, in the manuscript, we use the gendered term “Latino” when referring to cisgender LMSM. We did not use the gender-neutral term “Latinx” because of a lack of acceptance by the community and the term being considered a form of linguistic imperialism [30]. In its place, we use the gender-neutral and Spanish-affirming term “Latine” when referring to the broader Latin-American population [31].

## Methods

The study was approved by the Institutional Review Board of the University of California, Los Angeles (IRB#23–000550). Oral consent was obtained from all individual participants included in the study.

### Recruitment and study sample

LMSM were recruited from May 31, 2023, to March 6, 2024, to participate in four focus groups. Participants were recruited from six different cohorts of a LMSM-centered intervention focused on educating LMSM about different topics related to HIV prevention, including daily oral and LAI PrEP. Intervention participants comprised a diverse group of LMSM originally recruited from social media platforms (i.e., Facebook, Instagram), internal referrals from LGBTQ community-based organizations, and sexual hookup apps (i.e., Grindr and Growlr). Individuals were eligible to participate in the focus groups if they met the following criteria: self-identify as Latino, cisgender man, aged 18 years or older, reported engaging in anal or oral sex with another cisgender man in the past 24 months, were HIV-negative or status unknown, and were a resident of Los Angeles or surrounding counties. English language proficiency was not an inclusion criterion, as it was implied due to the recruitment of participants from an English language HIV prevention intervention. Further, it is possible that our study participants may differ somewhat from the broader LMSM population because they were originally recruited to participate in a multiple group session intervention and not a one-time focus group. Thus, intervention participants may have had more interest in learning about and discussing HIV prevention options than the broader LMSM population.

### Data collection

The primary aim of the focus groups was to elicit suggestions from LMSM on campaign content (i.e., messaging and imagery) to promote awareness of LAI PrEP among LMSM. In addition, participants were asked to describe: 1) their thoughts about the inclusion of a syringe in a LAI PrEP campaign; 2) the cultural values of the Latine community that should be reflected in a LAI PrEP campaign for LMSM; 3) the best messengers to promote LAI PrEP among LMSM; 4) ideal locations or venues to enhance access to a campaign developed for LMSM.

Prior to the start of each focus group, participants received a study information sheet and provided verbal consent over Zoom, which was witnessed by a co-facilitator and documented in a consent tracking log. Participants were then asked to complete a short demographic questionnaire. Each focus group began with a brief overview of LAI PrEP (i.e., efficacy, administration, side effects) to ensure that all participants had the same information about this new prevention option. Focus groups were conducted via Zoom, audio recorded, and lasted approximately 90 minutes. Participants received a $75 electronic Visa gift card for their participation.

### Data analysis

Focus group recordings were transcribed verbatim and reviewed for accuracy. Coding reliability thematic analysis (TA) was used to analyze the data [32]. To ensure accurate and reliable coding, an initial codebook was developed through an iterative process and consisted of deductive codes from the focus group guide and inductive codes from a line-by-line review of the transcripts. The starting point for the codebook was the deductive codes developed from the interview guide. These codes were supplemented with inductive codes drawn from the line-by-line review of the transcripts, filling a gap in the codebook.

The study team applied this initial set of codes to one transcript and then met to refine code descriptions, add/delete codes, and identify exemplar quotes associated with each code. Through group consensus, the team resolved coding discrepancies that emerged among coders to ensure consistent interpretation of the codebook. Next, two coders (O.N. and M.S.) uploaded the transcripts into Dedoose (a qualitative data management software) and performed a test of intercoder reliability using an additional transcript [33], resulting in a Cohen’s Kappa score of 0.87%, indicating near-perfect coder agreement [34].

The coded data were then organized into topic areas drawn from existing literature on key elements of public health campaigns that included: 1) content (i.e., messaging and imagery), 2) tone, 3) representation, and 4) accessibility [21,35]. The study team grouped related data within each topic area and constructed themes to highlight important considerations for developing a LAI PrEP campaign for LMSM. Themes were developed based on the frequency and/or depth of the related discussion in each area. The study team worked to ensure that the themes accurately reflected the dominant perspectives of participants within a given topic area and refined the themes accordingly.

## Results

In total, 30 LMSM participated across the four focus groups, with 5–9 participants per group. Table 1 includes participant demographic and select PrEP use characteristics. The average age of participants was 34 years (range = 18–57), and the majority identified as gay (77%), had obtained some college education (73%), and had some form of health insurance (90%). Among participants, PrEP use varied, with the majority (57%) having never used PrEP before and only 23% currently using the medication. All participants using PrEP were taking the daily oral pill.

**Table 1:** Participant demographic characteristics (N = 30).

Variable		N (%)^1^ or Mean, Range
*Demographic Characteristic*		
**Age (N = 30)**		Mean = 34 Range = 18–57
**Race**		
	White	11 (37%)
	Black or African American	0 (0%)
	Bi/Multi-racial	1 (3%)
	Other Race^2^	18 (60%)
**Sexual Orientation**		
	Gay	23 (77%)
	Bisexual	4 (13%)
	Queer	2 (7%)
	Other^3^	1(3%)
**Education**		
	Never attended school	0 (0%)
	Less than high school	0 (0%)
	High school/ GED	7 (23%)
	Associate degree	11 (37%)
	Bachelor’s degree	10 (33%)
	Graduate degree	1 (3%)
	Other	1 (3%)
**Employment status**		
	Unemployed	7 (23%)
	Working full-time	16 (53%)
	Working part-time	5 (17%)
	Retired	1 (3%)
	Other^4^	1 (3%)
**Income**		
	$0-$9,999	7 (23%)
	$10,000-$19,999	4 (13%)
	$20,000-$39,999	4 (13%)
	$40,000-$59,999	8 (27%)
	$60,000-$99,999	6 (20%)
	$100,000 or more	1 (3%)
**Health insurance**		
	Uninsured	3 (1%)
	Medi-Cal/Medicaid	13 (43%)
	Employer-provided insurance	9 (30%)
	Insurance through parent	2 (7%)
	Insurance through partner	1 (3%)
	Self-purchased health insurance	1 (3%)
	Other^5^	1 (3%)
**Ever used PrEP**		
	Yes	13 (43%)
	No	17 (57%)
**Currently using PrEP**^**6**^ **(N = 13)**		
	Yes	7 (54%)
	No	6 (46%)
**Length of time on PrEP (in months)**^**6**^ **(N = 6)**		M = 10 Range = 1–48
**Type of PrEP**^**7**^ **(N = 7)**		
	Truvada	3 (43%)
	Descovy	4 (57%)

^1^Percentages were rounded to the nearest whole number and may not add up to 100%.

^2^Other race includes: “Hispanic,” “Spanish/Puerto Rican/Mexican,” “Mexican Boricua,” “Mexican/Indian,” “Spanish,” “Latino,” “Mexican,” and “Mexican American”.

^3^Other orientation includes: “Pansexual”.

^4^Other employment includes: “Student”.

^5^Other health insurance includes: “private insurance”.

^6^Only include participants who responded to the question.

^7^Only includes current PrEP users who responded to the question.

Thematic analysis, described above, was used to guide construction of relevant themes from the data for each topic area (i.e., content, tone, representation, and accessibility) of a LAI PrEP campaign for LMSM. Themes generated included: the need to include basic information about LAI PrEP and to highlight its benefits; mixed preferences for including an image of a syringe; the need to incorporate positive messages/imagery; communicate the seriousness of HIV without stigmatizing, blaming, or scaring LMSM; the importance of incorporating Latine family members or chosen family/friend groups; the significance of involving Latine people in the promotion of LAI PrEP to LMSM; and creating access to a LAI PrEP campaign for LMSM. These themes are further described below.

### Content

#### The need to include basic information about LAI PrEP and to highlight its benefits.

Participants felt it was important to share basic information in a campaign to inform LMSM about LAI PrEP. This would include information about cost, access, and the effectiveness (i.e., efficacy) of LAI PrEP. As one participant noted, it is important to communicate that “*[LAI PrEP is] just as effective as pills*” (P10, Age 39, FG 2). However, some participants felt that specific information on side effects should be shared sparingly. They felt it was important to limit this information to avoid dissuading LMSM from considering LAI PrEP. As one of the men noted, “*I’ve always been a believer of less is more. Because people tend to, if they see so much information, they just leave*” (P16, Age 54, FG 2). In general, an approach preferred by participants is to share succinct and relevant information about LAI PrEP.

Participants also thought it was important to highlight the benefits of LAI PrEP. For example, participants believed the messaging should underscore that receiving an injection once every two months is more convenient than having to remember to take a pill every day, particularly for people who are forgetful and/or have busy lives:

“Kind of along the lines of the fact that you might forget the pills, so here’s something simpler, something more simple, because our lives are hectic as it is with so many responsibilities. One less responsibility to worry about.” (P20, Age 38, FG 3)

#### Mixed preferences for including an image of a syringe.

Participants held opposing views about whether to include an image of a syringe as part of a LAI PrEP campaign. Some participants felt strongly about excluding an image of a syringe because they believed that people are afraid of needles and associate them with pain. As one participant emphasized: “*I mean, you see little kids, they cry when they see a needle. It’s just a universal feeling of pain and discomfort*” (P26, Age 31, FG 3). Others felt it would be unnecessary to include a syringe because the delivery method is implied in the name (i.e., *injectable* PrEP):

“I think people can be intelligent enough to understand that it’s a different delivery method and all that without having to actually show the needle.” (P10, Age 39, FG 3)

In contrast, other participants were in favor of including a syringe to reflect the reality of the delivery method, but suggested various alternatives that could be used in place of a real needle, as illustrated in the following two quotes:

“Maybe perhaps having an image that resembles a needle. So, maybe not specifically like a sharp little [needle], but make it cute, make it simple, and something more welcoming in a sense…” (P5, Age 19, FG 2)

“I don’t know if it’s real or not, but I think it is, it’s this imagery of this pinup girl riding a rocket and it’s from the fifties or something… But it’s maybe a model, like a buff guy or a drag queen or something, riding a huge needle and saying, ‘*Get PrEPared for this summer*.’” (P2, Age 29, FG 1)

These examples highlight the importance of addressing people’s discomfort with injections.

### Tone

#### The need to incorporate positive messages/imagery.

Participants suggested that the tone of LAI PrEP campaign messages should be fun and light-hearted. To catch and retain consumers’ attention, participants believed that the messaging should be humorous and witty, and it should avoid being overly negative:

“If it’s kind of funny, if it’s kind of witty, people are going to remember that…. They’ll tell their friend” (P26, Age 31, FG 3).

“I feel like every time there’s a gay ad, it’s always a negative ad or it’s like a sad ad. And it’s like, no, we should be happy. Yeah, it’s a very sad topic [HIV]... I could see why it’s not a happy topic, but I feel like we’ve got to start being more positive.” (P19, Age 18, FG 3)

Additionally, participants felt the messaging should be Latine-centered and empowering: *“I think we should just make sure the focus is on the Latino community, and if it empowers other communities, great, but the message has to always be for empowerment.”* (P26, Age 31, FG 3)

Finally, participants felt it would be important to incorporate sex-positive messages and imagery as part of a campaign. This would include utilizing attractive Latino men as part of the campaign and accompanying these visuals with playful messaging. As one participant noted, the campaign should include “*hot, sexy, Latino men. Something eye-catching. Maybe making out.* [With the tagline] ‘Let’s talk about sex’” (P11, Age 39, FG 2).

#### Communicate the seriousness of HIV without stigmatizing, blaming, or scaring LMSM.

Participants discussed the importance of using statistics in a campaign to inform and/or re-educate LMSM about the disproportionate impact of HIV in their community and in communicating the importance of PrEP in curtailing future HIV infections:

“Maybe a more serious one could be one with a disproportionate amount of Latino men effective [infected by] HIV compared to white guys. So, people can see Latinos are way more affected.” (P11, Age 39, FG 2)

“I would also show the statistics. So, Latino men compared to other races, how our infection level is higher than other non-Latino men. So, show them that we’re affected most, but there is a way to prevent that and to bring that number down with PrEP.” (P10, Age 39, FG 2)

In contrast, some participants warned against making comparisons with other risk groups because this could potentially stigmatize or blame LMSM for their high rates of HIV. This sentiment was captured in this quote:

“I would really just not do comparisons. I know sometimes studies, or some messaging comes out where it’s like, ‘Oh, Latino men are more likely than white men to blah, blah, blah,’ and I would stray from that messaging because I think we don’t want to promote someone’s doing something better than someone or someone’s doing something worse than someone.” (P26, Age 31, FG 3)

Another common observation made by participants was that a campaign should avoid using scare tactics, because, like making comparisons, this type of messaging could stigmatize LMSM and make them less receptive to LAI PrEP messaging:

“You want to make sure that you don’t use fear tactics like, ‘Oh, if you don’t want to have AIDS, get PrEP.’” (P30, Age 39, FG 4)

“Don’t make the target wrong, bad, or awful… ‘If you don’t do this, this is going to happen to you.’ And there’s some messages out there that, ‘Have you tried this? Why not? Do you want to see this happening to you?’ That kind of approach. And you see that all the time, the scare tactic. And I think people just shut down when you approach it [that way], especially when you’re dealing with this kind of subject [HIV]. (P28, Age 56, FG 4)”

In summary, participants suggested that care must be taken when using statistics or comparisons with other groups to educate LMSM about HIV so as not to cause stigma, blame, or fear in the population.

### Representation

#### The importance of incorporating Latine family members or chosen family/friend groups.

In discussing representation, participants stressed that a LAI PrEP campaign should incorporate cultural values that are important to Latine people (e.g., being family oriented). Much of the discussion centered on having family members (e.g., mothers, grandmothers, *chismosa tías* [gossipy aunts]) included in a campaign showing love, support, and concern for the safety of their gay Latino family members:

“And then, including maybe a family element kind of like, hey, you’re going home, and then your mom or your family sees that you’re home safe, about safety and I guess home life, and people that have your back showing a lot of love.” (P20, Age 38, FG 3)

By incorporating a familial element, participants believed it could help normalize gay identity within Latine families: “*Make it [a campaign] include being part of families and stuff like that. Normalize being gay in a Latino family. It’s pretty hard, but we have to do it*” (P10, Age 39, FG 2). In addition, participants felt a LAI PrEP campaign could specifically depict a gay *tío* [uncle] supporting his younger nieces/nephews (regardless of their sexuality) and acting as a source of information about available HIV prevention options:

“It’s always the [gay] *tío* [uncle], right? So even that part, even seriously having that uncle with the niece or the nephew, that’s one way of tying it in because it is true. It’s always the uncle who supports the younger generation, whether they’re straight, gay, or not. So, I like that format. It’s really family-oriented because Latinos are family-oriented. So that would be a sweet touch or a sweet roundup to that.” (P29, Age 46, FG 4)

However, participants noted that some Latine gay people are estranged from their families of birth and might not relate to these familial images. As an alternative, a LAI PrEP campaign can choose to represent one’s chosen family (e.g., romantic partners and/or friends), which would still reflect the family values (e.g., unity, solidarity, support):

“I know in the representation of what’s meant to be Latino, I know we talked about the family-oriented, but there’s also individuals who have been sort of cast out from their family because they’re gay. So maybe incorporate, it’s not only your blood related family, but also the family that you are creating with your friends, or I don’t know, maybe a partner or whatever.” (P30, Age 39, FG 4)

#### The significance of involving Latine people in the promotion of LAI PrEP to LMSM.

Participants emphasized that the messengers (i.e., the “face”) of a LAI PrEP campaign should be members of the Latine community. By including Latine people as messengers, participants believed it would enhance the credibility, relatability, and authenticity of a campaign: “*If they’re part of the community and they’re endorsing it, then it gives the program credibility*” (P28, Age 56, FG 4).

Among the various groups mentioned, Latine celebrities (e.g., musicians, social media influencers, drag queens) were thought to be potentially effective messengers because they have a large, dedicated audience and can influence their followers. This point is demonstrated in the following two quotes:

“I remember reading online that certain grandmas became accepting to the LGBT people because Bad Bunny said he was bi. He definitely has sway just because he’s such a big singer.” (P1, Age 26, FG 1)

“Well, just drag Queens [Bianca Del Rio], like RuPaul’s Drag Race. It’s a very big platform, very widely watched.” (P27, Age 38, FG 1)

In contrast, some participants had trouble relating to celebrities and felt LMSM would be more receptive to seeing “everyday” Latinos reflected in a LAI PrEP campaign. As one participant describes:

“I personally don’t look at stuff that’s being promoted by an actor. What catches my eye is regular people up there, like a Latino gay man. It doesn’t have to be an actor. I think relatability is important. I know that for a lot of our community, we listen to a lot of people that are icons. But also, because it’s for the people, and I personally would react more to just an ordinary person up there promoting.” (P16, Age 54, FG 2)

They also believed it would be important to depict diverse Latine people in a campaign (e.g., skin tone, body type, gender expression, age, nationality, and occupations). An additional suggestion is to have LMSM share their personal journey in using LAI PrEP to help encourage other LMSM to consider using this option.

As a final point in representation, participants felt that politicians and medical providers can also serve as effective messengers, particularly if they identify as Latine and gay:

“We have a lot of openly gay officials in Southern California. One of the council members in Huntington Park is openly gay and I know that he’s highly involved in the community.” (P27, Age 28, FG 4)

“I feel like a medical provider or someone who works in the medical industry would be a good source to add an authenticity to it. I think maybe it would be helpful if it would be someone that identifies as Latino and queer and also works as a doctor….” (P24, Age 24, FG 3)

### Accessibility

#### Creating access to a LAI PrEP campaign for LMSM.

As a last point of discussion, participants felt that a LAI PrEP campaign should be made accessible to our diverse LMSM community. One strategy to enhance accessibility is to develop campaign materials in both Spanish and English:

“[Make] sure that it’s bilingual, that not only the English-speaking people can understand, but also Spanish-speaking people, because we... In Los Angeles, there is a lot of Latinos from all different cultures and sometimes, unfortunately, we do not read English.” (P29, Age 46, FG 4)

Another strategy is to concentrate outreach efforts in predominantly Latine spaces: “*I think like using billboards where there’s heavier Latin communities, in the Valley, would be effective*” (P11, Age 39, FG 2). Participants also suggested using social media platforms (e.g., TikTok, Facebook) and gay hookup apps (e.g., Scruff, Grindr) to increase access to a LAI PrEP campaign, as one participant explained:

“Social media has been going popular right now…. And on TikTok, being able to promote, talk about PrEP, and maybe doing small commercials, or interviews is a way to be able to promote PrEP.” (P18, Age 22, FG 2)

Together, having bilingual content displayed in relevant geographic and digital locations would improve access to a LAI PrEP campaign targeted to LMSM.

## Discussion

The findings from this study offer several practical recommendations for developing a campaign to increase awareness of LAI PrEP among LMSM in a high HIV prevalence jurisdiction that is a priority area in the Ending the HIV Epidemic in the US initiative. Using a qualitative approach, seven themes were constructed from the data that offer suggestions for essential components of a LAI PrEP campaign tailored for LMSM including campaign content, tone, representation, and accessibility. Below we discuss the themes considering the existing literature on informing, developing, and evaluating public health and HIV campaigns for racial/ethnic sexual minority men. Table 2 provides a summary of key campaign elements to include in a LAI PrEP campaign for LMSM.

**Table 2:** Key elements to include in a LAI PrEP campaign for LMSM.

• Provide basic information on LAI PrEP (e.g., cost, accessibility, and effectiveness) and where it can be accessed
• Highlight the benefits of LAI PrEP (e.g., convenience, greater discretion).
• Include positive, uplifting, or empowering imagery and messaging
• Educate LMSM about the current impact of HIV on their community without stigmatizing, blaming, or scaring them
• Include imagery of family as support systems for using PrEP
• Include LMSM as messengers in a campaign
• Incorporate personal testimonials from LMSM who are using LAI PrEP
• Ensure promotional materials are available in both English and Spanish
• Place billboards/advertisements in neighborhoods with a high concentration of Latine people
• Utilize relevant social media platforms (e.g., TikTok, Facebook) and gay hookup apps (e.g., Scruff, Grindr) to reach LMSM

Participants indicated that a key component of a LAI PrEP campaign for LMSM is the inclusion of basic information about the product and highlighting its benefits for HIV prevention. Participants believed that providing basic information will help ensure an understanding of and build interest in LAI PrEP among LMSM. A similar finding was noted among Black MSM [26]. Public health campaigns seeking to promote interest in LAI PrEP should highlight its benefits as a new prevention strategy. This includes underscoring that LAI PrEP may be a more appropriate option for MSM who struggle with taking daily pills [36–39]. A campaign may also want to highlight LAI PrEP’s social benefit of reducing the potential for gay and/or PrEP-related stigma that can result from being seen picking up a PrEP prescription from a pharmacy or someone inadvertently seeing an individual’s PrEP medication bottle [38,40]. If the goal of a LAI PrEP campaign is to increase awareness and cultivate interest, it is essential that basic information be part of the campaign, including information on where to access LAI PrEP locally.

Participants held conflicting views about the inclusion of a syringe in a LAI PrEP campaign. Those in favor of displaying a syringe suggested doing so to represent how LAI PrEP is administered to patients. However, even these suggestions were not to include an image of an actual needle, but instead to use a playful or cartoonish image in its place. In contrast, some participants felt that including an image of a syringe in a public campaign would cause viewers to immediately think of the pain normally associated with receiving an injection and would discourage interest in the product. This view aligns with findings from prior work. For example, the most common fear and anxiety associated with healthcare procedures are needles and pain [41,42]. According to the CDC, viewing a picture of needles can be enough to induce fear in some people [43]. A fear of needles often originates in childhood but can continue into adulthood [42]. Persons with an intense fear of needles are considered to have trypanophobia. This fear can be so extreme that some people may avoid receiving life-saving medications such as vaccines [43]. Developers of a LAI PrEP campaign may want to consider using alternative visual imagery in place of depicting a realistic syringe to avoid potentially triggering those with trypanophobia. This can include utilizing images that symbolize protection (e.g., a shield), are empowering (i.e., depicts smiling people who just received an injection), or are cartoonish in nature (e.g., the bandage emoji). Given the strong association between viewing needles and fear, LAI PrEP campaigns should avoid including any realistic images of needles.

Participants suggested two distinct tones they wanted to see conveyed in a LAI PrEP campaign. First, they underscored a need to ensure that messages and imagery are positive and uplifting. Second, participants suggested that a LAI PrEP campaign should include information about the disproportionate impact of HIV on LMSM but without stigmatizing, blaming, or scaring the population. Further, it is important that HIV prevention campaigns centered on LMSM should seek to reduce the existing HIV stigma within the Latine community and increase acceptance of Latino gay men. Prior research with Black MSM has found that some HIV prevention campaigns that include a monolithic representation of BMSM is stigmatizing to BMSM because it suggests that racial/ethnic sexual minority men are the only groups impacted by HIV [22,25,27]. For a LAI PrEP campaign, one approach to reducing stigma is to include a diverse representation of LMSM (e.g., age, body type, skin tone, gender expression) and avoid depicting stereotypical images (e.g., muscular men in speedos).

In this study, the concept of representation was identified as an essential piece of creating a culturally appropriate LAI PrEP awareness campaign. The suggestion of participants to incorporate Latine family members or chosen family/friend groups in a campaign represents the idea that family is central in Latine culture, be it immediate or extended family or close friends [44]. In prior research on preferences for online advertising of smoking cessation programs for Latino smokers, participants preferred ads utilizing the cultural value of family (*familismo*) over fatalism (*fatalismo*) [45]. As for the promotion of a LAI PrEP campaign, participants strongly endorsed the idea that messengers be Latine people to add authenticity to a campaign. They offered a variety of suggestions of messengers that included Latine celebrities, public figures, influencers, medical providers, and everyday Latine people who looked like them. Additionally, participants suggested that personal stories of LMSM using LAI PrEP may be an effective strategy for sharing information about this prevention strategy and encouraging its use among LMSM. These personal testimonials can tap into the emotions of LMSM. In prior research with diverse groups of MSM, personal testimonials have been suggested as a strategy for increasing PrEP uptake [46,47].

A main tenet of representation is that a campaign for LMSM resonates with and engages the population, and that it reflects Latine people, including LMSM. In prior LMSM focused campaigns, cultural relevance was achieved through deep community involvement [48–51]. This included working with community to develop messaging that reflected current beliefs and the lived experiences of Latino MSM [49–51]. However, previous campaigns were sensitive to the reality that some LMSM may not openly identify as gay or recognize same-sex behavior [48,49]. In addition, cultural norms were represented through the use of visuals familiar to the community (e.g., parks), and by having real LMSM (i.e., not actors) present Spanish, Spanglish, or bilingual content to viewers [48,51]. In addition, stigma reduction was accomplished in several studies by bundling HIV testing with other general health screenings (e.g., diabetes, hypertension), using humor, empowering the population, and representing family values [48,49]. Another example of a campaign that avoided stereotyping framed HIV prevention not as a corrective behavior but rather as a form of self-care for LMSM and a decision that reflects their familial responsibility [50]. It is important that representation in a LAI PrEP campaign be acceptable among the population it is trying reach.

Participants offered multiple strategies for enhancing access to a LAI PrEP campaign for LMSM. The prime among them was using popular social media platforms (e.g., Facebook, Instagram, X [Twitter], TikTok). These platforms have been shown to be effective in promoting PrEP awareness and uptake among LMSM [52,53]. In preparing to promote a LAI PrEP campaign, it will be important to remember that social media consumption is rapidly changing and that some previously popular platforms may lose relevancy with today’s consumers (e.g., X) [54]. As such, those developing a LAI PrEP campaign would benefit from seeking counsel from community members or a community advisory board comprised of members of their priority populations to assess which platforms are still relevant to the populations they are aiming to reach. An additional strategy offered by participants to enhance access is the placement of physical advertisements in neighborhoods with a high concentration of Latine people. This finding is consistent with prior literature that evaluated the impact of PrEP social marketing campaigns in Latine communities [55,56]. Finally, developing bilingual or exclusively Spanish language campaign materials is important for ensuring access among Latine persons with limited English proficiency or who are monolingual Spanish speakers. Appropriately using Spanish and/or English language is a way to make LMSM with language discordance feel included in a LAI PrEP campaign [57].

### Limitations

The findings should be interpreted considering the study limitations. By recruiting participants from an existing HIV prevention intervention for LMSM in Los Angeles, our findings may not be transferable to other groups of LMSM who reside outside of Los Angeles and surrounding counties. In addition, one of the drawbacks of conducting a focus group is that a few voices may dominate the conversation, limiting other participants from expressing divergent or unique views and potentially leading to group conformity around issues being discussed. Similarly, all focus groups were conducted in English, and, as such, our findings may not reflect the perspectives of monolingual Spanish speaking LMSM in Los Angeles. To better reflect the diversity of the LMSM population, future research should be conducted with monolingual Spanish speaking LMSM that includes both individual in-depth interviews and group perspectives captured in focus groups. An additional limitation is the lack of inclusion of gender diverse populations (e.g., transgender, nonbinary) given high rates of HIV [58]; future research should explore LAI PrEP messaging among these populations.

## Conclusion

Increasing PrEP uptake, including LAI PrEP, is a critical piece of our effort to bring an end to HIV in the US. A future LAI PrEP campaign developed for LMSM must reflect the language, values, and perspectives of the local community to ensure greater engagement and follow-through behavior (i.e., talking with a provider about LAI PrEP). Without this local perspective, a campaign will not resonate with or have meaning for its viewers. The recommendations offered in the current study could be repeated with future long-acting injectable PrEP products to establish culturally appropriate campaigns for LMSM of these new products.

## Supporting information

S1 File Raw anonymous dmeographic data file. (XLSX)
